# Refugee-like migrants have similar health needs to refugees: a New Zealand post-settlement cohort study

**DOI:** 10.3399/bjgpopen20X101013

**Published:** 2020-02-19

**Authors:** Jonathan Donald Kennedy, Serena Moran, Sue Garrett, James Stanley, Jenny Visser, Eileen McKinlay

**Affiliations:** 1 Senior Lecturer, Department of Primary Health Care & General Practice, University of Otago; General Practitioner, Newtown Union Health Service, Wellington, New Zealand; 2 Clinical Research Nurse, Department of Primary Health Care & General Practice, University of Otago; Registered Nurse, Newtown Union Health Service, Wellington, New Zealand; 3 Research Fellow, Department of Primary Health Care & General Practice, University of Otago, Wellington, New Zealand; 4 Associate Professor, Biostatistician, Department of Primary Health Care & General Practice, University of Otago, Wellington, New Zealand; 5 Senior Lecturer, Department of Primary Health Care & General Practice, University of Otago, Wellington, New Zealand; 6 Associate Professor, Department of Primary Health Care & General Practice, University of Otago, Wellington, New Zealand

**Keywords:** refugees, human migration, primary health care, delivery of health care, New Zealand

## Abstract

**Background:**

Refugees and asylum seekers have specific health and social care needs on arrival in a resettlement country. A third group — migrants with a refugee-like background (refugee-like migrants) — are less well defined or understood.

**Aim:**

Using routinely collected data, this study compared demographics, interpreter need, and healthcare utilisation for cohorts of refugee-like migrants and refugees.

**Design & setting:**

A retrospective cohort study was undertaken in Wellington, New Zealand.

**Method:**

Data were obtained for refugee-like migrants and refugees accepted under the national quota system (quota refugees), who enrolled in a New Zealand primary care practice between 2011 and 2015. Data from the primary care practice and nationally held hospital and outpatient service databases, were analysed. Age and sex standardisation adjusted for possible differences in cohort demographic profiles.

**Results:**

The cohorts were similar in age, sex, deprivation, and interpreter need. Refugee-like migrants were found to have similar, but not identical, health and social care utilisation to quota refugees. Primary care nurse utilisation was higher for refugee-like migrants. Clinical entries in the primary care patient record were similar in rate for the cohorts. Emergency department utilisation and hospital admissions were similar. Hospital outpatient utilisation was lower for refugee-like migrants.

**Conclusion:**

This research suggests that health, social care, and other resettlement services should be aligned for refugee-like migrants and quota refugees. This would mean that countries accepting quota refugees should plan for health and social care needs of subsequent refugee-like migrant family migration. Further research should investigate matched larger-scale national health and immigration datasets, and qualitatively explore factors influencing health-seeking behaviour of refugee-like migrants.

## How this fits in

Refugees are recognised to have high health and social care needs. When refugees arrive through quota systems in a country, family members (refugee-like migrants) are likely to follow. This research found that refugee-like migrants have similar or greater health utilisation to refugees. Therefore, refugee-like migrants also need health and social care services that are systematically planned and delivered.

## Introduction

Discourse about global migration often separates people into those who leave their country of origin by choice — migrants — and those forced to leave for their own safety — refugees.^1^ To facilitate protection from persecution, the United Nations Convention in 1951,^2^ supported by the Protocol in 1967,^3^ developed the official definition for refugees and in 2016 37 countries, including New Zealand,^4^ accepted refugees within refugee resettlement quota programmes (quota refugees).^5^ However, people may fall into a grey area between choice and being forced, with migration influenced by means and opportunity to overcome fluctuating local and international political and social barriers.^1^ Many people try to migrate as asylum seekers,^6^ as with the major contemporary movements of people into Europe from conflict zones in the Middle East and Africa, supported by international frameworks, and in some cases by national legislation; for example, as in New Zealand^4^ and the UK.^7^ Others use legal migrant categories such as those designed to facilitate family reunification.^8^ Health utilisation characteristics of legal migrants with refugee-like backgrounds (refugee-like migrants) are not well described in the literature, and it is this group that this research addresses.

Refugees, including quota refugees, have significant health and social needs,^9^ which impact on quality of life, economic contribution, and health and social service utilisation.^10–15^ When quota refugees arrive in a country, they can access a range of state-sponsored social support and health services, including social work, health screening, and psychological services.^16,17^ In New Zealand, with an annual quota of 1000 refugees,^4^ this is initiated at a 6-week residential orientation programme in Auckland.^18^

An early priority for quota refugees is to sponsor reunification of family members still resident in their country of origin or a country of refuge.^19^ In New Zealand, half of former refugees apply to sponsor family members to migrate to New Zealand, and two-thirds of applications are successful.^20^ Most of these refugee-like migrant family members have access to publicly funded health care, but some arrive in New Zealand under work or visitor visa categories^21^ without access to publicly funded health services. There is no systematic state-sponsored settlement support or health screening offered to the refugee-like migrant group and no comprehensive data is routinely collected about their health status or needs. However, healthcare access by different groups of people with refugee-like backgrounds in New Zealand could be expected to be strongly influenced by the national (usual care) healthcare programmes that are offered to them. Table 1 gives New Zealand contextual information about these groups of people.

**Table 1.** Terms and New Zealand background information

	**Visa categories**	**Healthcare entitlement**	**Health screening and systematic support**	**Annual intake**
**Quota refugee**	Granted permanent residence status in New Zealand.	Eligible for fully funded health services.	Offshore health screening.Six-week residential orientation and additional health screening at Mangere Refugee Resettlement Centre.^22^Settlement and social care support in community.	750 per annum during the period of this research, since raised to 1000 per annum in July 2018, and planned to increase further to 1500 per annum in July 2020.^23^Countries of origin have varied over time according to government priorities.^24^
**Refugee-like migrant**	Heterogenous group with variety of New Zealand visa categories; for example, refugee family support category,^23^ visitor visa, or work visa categories.	Variable, some are granted New Zealand permanent residence status and have access to fully funded health services, others on visitor visa or work visa for less than 2 years have no funded health or social care support.	Variable offshore health screening depending on visa category.Arrive to family members in community.Additional health screening not systematically offered.	Limited information.
**Asylum seeker (convention refugee)^23^**	May apply for New Zealand Permanent Residence status if granted ‘refugee or protected person’ status at conclusion of asylum seeker application process.	Fully funded health services while application being processed.	Variable offshore health screening depending on initial visa category.Optional additional health screening funded on arrival once asylum seeker application lodged.No systematic social care or settlement support.	Variation in applications, from 333 people in 2010–2011 to 510 people in 2018–2019.^25^

The UK, which has a health system with similarities to New Zealand’s, also accepts refugees through resettlement schemes. The UK is planning for an additional 5000 quota refugees in 2020–2021 through a new resettlement scheme, which consolidates the Vulnerable Persons’ Resettlement Scheme, the Vulnerable Children’s Resettlement Scheme, and the gateway protection programme.^26^ Therefore, the UK will likely also experience differences between health and social care needs for quota refugees, and migrants with refugee-like backgrounds.

The aims of this project were to compare, with participants selected from a primary care context, characteristics and needs of refugee-like migrants and quota refugees*,* by examining routinely collected data on demographics, interpreter need, health utilisation, health screening, and immunisation.

### Newtown Union Health Service

Since 2003, Newtown Union Health Service (NUHS), a Wellington not-for-profit primary care service, received public funding to provide primary care for regional refugee arrivals.^27^ Since October 2009, the service routinely identified refugee-like migrants according to the following definition: 'From a background comparable to people admitted to New Zealand with refugee status and has similar health needs and requires screening similar to a refugee.'^28^ Examples accompany the definition to guide clinicians in classification decisions. Quota refugee and refugee-like arrivals received oversight of their enrolment and initial health care by a service-based ‘refugee team’ consisting of a GP, a primary care nurse and a social worker. Six-monthly reporting to health authorities was required, and team members audited service data to maintain quality; for example, patients joining the service who had been resident in New Zealand or other developed countries for long periods were not included as refugee-like migrants*,* as they were not considered to meet the ‘health need’ part of the clinical definition.

Services provided to both quota refugees and refugee-like migrants at NUHS included an initial ‘Well Health Check’, health screening for acute and chronic illnesses, catch-up immunisations, referral to appropriate psychological services, support with accessing social care, and ongoing routine primary care, with no-cost access to professional interpreters for consultations.

In New Zealand primary care is part publicly funded, with variable patient fee-for-service payments.^29^ At NUHS, consultation fees were the same for both quota refugees and refugee-like migrants with a small higher fee difference for patients attending GP-led versus nurse-led clinics.

The service had more than 1200 patients with a quota refugee or refugee-like migrant background; that is, 25% of the practice patient population. The service enrolled on average 60 quota refugees each year over the 5-year time period to 2015, constituting 8% of New Zealand’s annual arrivals (approximately 750 quota refugees). The service first received refugees in 1987, with arrivals from Cambodia and Vietnam, followed by large groups from Iraq, Ethiopia, Somalia, and Afghanistan,^27^ and more recently Colombia, Burma, and Syria, following national trends. It had former refugees from more than 25 different ethnic and language groups.

No other general practices to the authors' knowledge systematically identified a refugee-like group, making this an opportunity, not likely to be available in other primary care settings, to examine health characteristics of an important subset of migrants for planning and service delivery purposes.

## Method

### Study design

This retrospective cohort study used routinely collected and anonymised primary and secondary care data, matched using National Health Index (NHI) number, a unique identifier assigned to every person using health and disability support services in New Zealand.^30^ Data were obtained from a primary care practice (NUHS), which had collected data for both refugee-like migrant and quota refugee groups.^27^

### Participants

Participant selection was based on the date refugee-like migrant and quota refugee arrivals were first entered into the NUHS patient management system (PMS), and included all arrivals from 1 January 2011 to 31 December 2015. Quota refugees were identified based on information supplied by the New Zealand Red Cross. Refugee-like migrants were identified and routinely classified by a practice-based refugee team consisting of a GP, primary care nurse, and social worker, based on information supplied by the patient and their families. Follow-up time was calculated from date of entry into the PMS to the end of the study period.

### Variables

Primary care data were extracted from the PMS. These included NHI number, age, sex, self-reported ethnic group, socioeconomic deprivation, and health screening records. Primary care utilisation data were derived from service invoice records. Events where staff entered clinical notes in the primary care patient record, designated as ‘encounters’, included all consultations with clinical staff, phone contacts, and discrete clinical notes. These were extracted using the PMS audit function. National datasets held by the Ministry of Health provided hospital admissions, outpatients and emergency department visits, maternity visits, mental health outpatient attendance, and immunisation records, with linking by participant NHI numbers. Breast screening was defined as completed mammography, cervical screening was cervical cytology testing offered to patients, stool testing was for faecal parasites, tuberculosis screening was as directed by public health units (tuberculin skin test, interferon-gamma release assay, and/or chest X-ray).^31^ Blood tests were for a range of long-term conditions and conditions appropriate to country of origin as directed by a national refugee screening protocol.^32^ External primary care visits (such as to after-hours services) were obtained from regional primary health organisation data.^33^ Utilisation data were collected from 1 January 2011 to 31 December 2016.

Ethnicity was reported in detail and aggregated into four broad groups based on New Zealand standard ethnicity coding practice.^34^ Small-area socioeconomic deprivation using NZDep 2013 quintiles^35^ was based on geo-coded address at the time that study data were extracted from the PMS.

### Analysis and statistical methods

Patient sociodemographic and health profiles at baseline were described as frequencies and percentages, with 95% confidence intervals (CI). Participation in screening activities was reported as a proportion of individuals screened, with 95% CI. Rates of health service utilisation were reported as rates per 100 person-years of follow-up time with 95% CI, and by mean per 100 persons per annum with 95% CI for longitudinal figures. For longitudinal analysis of utilisation, event counts were grouped by each year after initial enrolment with denominators determined by person-time summaries by year (for example, in first year post-enrolment; in second year post-enrolment). Rates of health service utilisation were age- and sex- standardised to improve comparability of results between the two groups (standardised to the World Health Organization age standard,^36^ with sex weighting achieved by splitting weights equally into males and females). Maternity-related rates were calculated for women aged 15–54 years (inclusive) at the start of the study period. Screening rates for breast and cervical screening offered were calculated for eligible women aged 45–69 years and 20–69 years respectively (New Zealand has since changed cervical screening guidelines to recommend screening for women aged 25–69 years).^37^ Stool testing, tuberculosis screening, and blood tests rates were calculated for all arrivals. Analysis was conducted in Excel and R (version 3.5).

## Results

### Participants

A total of 235 refugee-like migrants and 346 quota refugees were identified from the pre-existing practice classifications in the 5-year eligibility period. From these, exclusions were made of three refugee-like migrants (owing to substantial time spent in other developed countries before migrating to New Zealand), four quota refugees (previously enrolled in practice prior to the study period and re-enrolled during the study period), and five quota refugees (who were asylum seekers incorrectly recorded as quota refugees). Finally, three refugee-like migrants were reclassified as quota refugees following review of their records. The analysed cohorts included 229 refugee-like migrants and 340 quota refugees. Twenty-two quota refugees and 21 refugee-like migrants transferred out from the practice during the research period.

**Figure 1.**
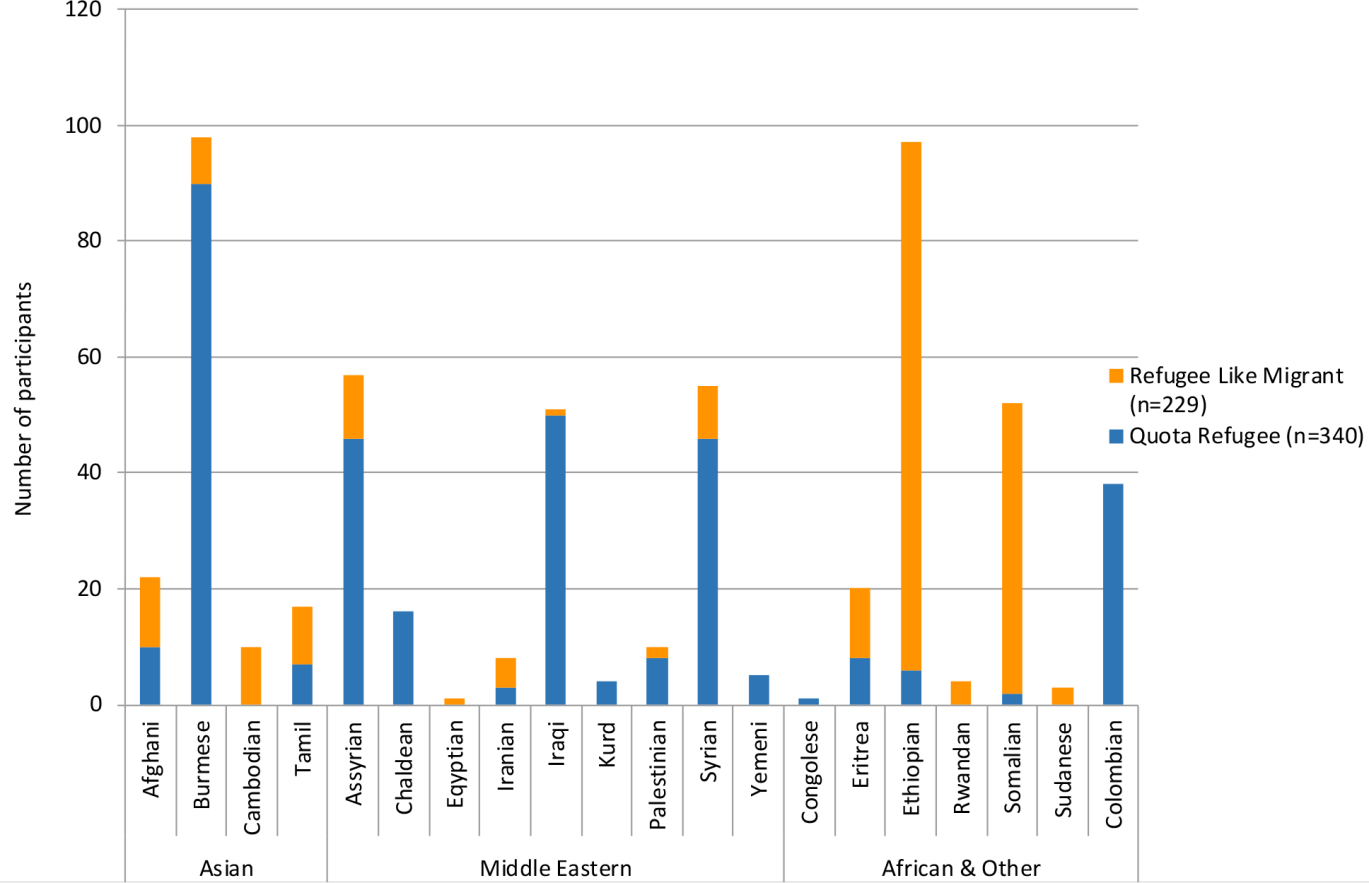
Refugee-like migrants and quota refugees by self-identified ethnic group

Table 2 and Figure 1 present baseline demographics for the cohorts. Participants in both cohorts entered the study throughout the study period, resulting in a range of person-years of follow-up. The cohorts were similar in demographic characteristics. Both were weighted towards younger age groups, particularly in the refugee-like migrant cohort, and were evenly split by sex. Refugee-like migrant and quota refugee ethnic groups reflected New Zealand refugee acceptance patterns,^24^ with more refugee-like migrants in earlier-established ethnic groups. This explains why more refugee-like migrants are from African countries, which were earlier focuses of New Zealand resettlement policy compared with more recent groups. Eighteen language groups were represented in the cohorts (data not shown). The majority of participants in both groups resided in the most deprived areas (quintiles 4 and 5).^35^ A large majority of both refugee-like migrants and quota refugees were recorded as needing a professional interpreter on arrival to the practice.

**Table 2.** Sociodemographic characteristics of refugee-like migrants (*n* = 229) and quota refugees (*n* = 340) at enrolment in practice

**Variable**	**Level**	**Quota refugee**	**Refugee-like migrant**
***n***	**% (95% CI**)	***n***	**% (95% CI**)
Age group (years)	0–9	41	12.1 (8.8 to 16.0)	21	9.2 (5.8 to 13.7)
	10–19	69	20.3 (16.1 to 25.0)	40	17.5% (12.8 to 23.0
	20–29	50	14.7 (11.1 to 18.9)	73	31.9 (25.9 to 38.3)
	30–39	74	21.8 (17.5 to 26.5)	50	21.8 (16.7 to 27.8)
	40–49	61	17.9 (14.0 to 22.4)	12	5.2 (2.7 to 9.0)
	50–59	23	6.8 (4.3 to 10.0)	17	7.4 (4.4 to 11.6)
	60–69	12	3.5 (1.8 to 6.1)	12	5.2 (2.7 to 9.0)
	≥70	10	2.9 (1.4 to 5.3)	4	1.7 (0.5 to 4.4)
	Median (IQR)	31 (16 to 43)		27 (19 to 36)	
Sex	Female	166	48.8 (43.4 to 54.3)	121	52.8 (46.2 to 59.4)
	Male	174	51.2 (45.7 to 56.6)	108	47.2 (40.6 to 53.8)
Ethnicity (grouped)	Asian	107	31.5 (26.6 to 36.7)	40	17.5 (12.8 to 23.0)
	Middle Eastern	178	52.4 (46.9 to 57.8)	29	12.7 (8.6 to 17.7)
	Latin American	38	11.2 (8.0 to 15.0)	0	0.0 (0.0 to 1.3)
	African	17	5.0 (2.9 to 7.9)	160	69.9 (63.5 to 75.7)
NZDep Quintile	1	1	0.3 (0.0 to 1.6)	5	2.2 (0.7 to 5.0)
	2	28	8.2 (5.5 to 11.7)	11	4.8 (2.4 to 8.4)
	3	23	6.8 (4.3 to 10.0)	33	14.4 (10.1 to 19.6)
	4	96	28.2 (23.5 to 33.3)	27	11.8 (7.9 to 16.7)
	5	183	53.8 (48.4 to 59.2)	151	65.9 (59.4 to 72.1)
	Missing	9	2.6 (1.2 to 5.0)	2	0.9 (0.1 to 3.1)
Interpreter needed	Yes	311	91.5 (88.0 to 94.2)	188	82.1 (76.5 to 86.8)
Years of follow-up data since enrolment	0 to <1	8	2.4	9	3.9
	1 to <2	101	29.7	31	13.5
	2 to <3	48	14.1	58	25.3
	3 to <4	74	21.8	59	25.8
	4 to <5	85	25.0	30	13.1
	5 to <6	24	7.1	42	18.3
	Median (IQR)	Median 3.2 (1.8 to 4.1)		Median 3.3 (2.2 to 4.4)	

CI = confidence intervals. IQR = interquartile range.

### Health resource utilisation, screening, and immunisation

Table 3 presents health utilisation, screening, and immunisation data for the quota refugee and refugee-like migrant cohorts. Primary care utilisation is presented by provider type, encounters, and visits to other primary care providers, including after-hours. Secondary care utilisation is presented by emergency department, inpatient, outpatient, maternity deliveries, and mental health care liaison contacts. Screening data are presented for arrival laboratory and tuberculosis screening, and for national breast and cervical cancer screening programme participation. Immunisation data are presented for immunisation initiation rates and timing, and the mean number of immunisation consultations for those immunised.

**Table 3.** Health utilisation, screening and immunisation by cohort adjusted for age and sex

**Group**	**Variable**	**Unit**	**Quota refugee**	**Refugee-like migrant**
**Crude rate**	**Adjusted rate with 95%** CI	**Crude rate**	**Adjusted rate with 95%** CI	**Adjusted relative risk RLM:QR with 95%** CI
**Primary** **c** **are** **u** **tilisation**	GP consultations	Rate per 100 person-years in study	256.1	278.7 (265.8 to 293.6)	225.6	255.9 (238.4 to 276.5)	0.92 (0.84 to 1)
	Primary care nurse consultations	Rate per 100 person-years in study	223.2	247 (234.6 to 261.3)	263.4	296.7 (276.4 to 320.1)	1.2 (1.1 to 1.31)
	Primary care social worker consultations	Rate per 100 person-years in study	18.0	23.7 (20.4 to 29.7)	60.8	42.3 (37.1 to 51.2)	1.78 (1.47 to 2.16)
	Encounters	Rate per 100 person-years in study	953.9	1035.1 (1009.3 to 1062.6)	966.2	1,057 (1019.3 to 1097.6)	1.02 (0.98 to 1.07)
	Visits to other primary care providers including after-hours	Rate per 100 person-years in study	14.4	15.5 (12.7 to 21)	10.0	15.7 (10.3 to 25.6)	1.01 (0.66 to 1.55)
**Secondary** **c** **are** **u** **tilisation**	Emergency department visits	Rate per 100 person-years in study	37.2	40 (34.4 to 48.1)	26.3	36.7 (28.7 to 48.7)	0.92 (0.7 to 1.21)
	Inpatient admissions	Rate per 100 person-years in study	29.8	34.1 (28.7 to 41.9)	24.4	30.4 (23.6 to 41.1)	0.89 (0.67 to 1.19)
	All outpatient visits	Rate per 100 person-years in study	254.0	300.6 (283.8 to 319.3)	168.5	208.0 (191.6 to 227.6)	0.69 (0.63 to 0.76)
	Outpatient dental visits	Rate per 100 person-years in study	40.5	48.7 (43.3 to 56.5)	22.5	22.3 (18.4 to 30.3)	0.46 (0.37 to 0.57)
	Outpatient allied health	Rate per 100 person-years in study	39.6	41.8 (37.3 to 48.8)	28.7	43.5 (36.6 to 54)	1.04 (0.85 to 1.27)
	Outpatient maternity	Rate per 100 person-years in study	75.0	75.5 (65.7 to 86.7)	85.1	89.3 (78.3 to 103.3)	1.18 (0.98 to 1.43)
	Maternity deliveries	Rate per 100 person-years in study	6.1	6.7 (3.9 to 11.2)	6.7	5.9 (3.5 to 12.5)	0.88 (0.45 to 1.74)
	Mental health care liaison	Rate per 100 person-years in study	57.4	54.1 (49.2 to 61.2)	32.0	18.9 (16.3 to 25.9)	0.35 (0.3 to 0.41)
**Screening**	Blood	Percentage of cohort	86.5	86.9 (75.8 to 100.0)	79.0	78 (64.2 to 100.0)	0.9 (0.71 to 1.13)
	Stool	Percentage of cohort	95.3	95.9 (84.1 to 100.0)	81.7	83 (67.2 to 100.0)	0.87 (0.68 to 1.1)
	Tuberculosis	Percentage of cohort	87.9	89.2 (77.8 to 100.0)	57.6	58.1 (44.8 to 80.5)	0.65 (0.49 to 0.86)
	Cervix^a^	Percentage of cohort	94.9	94 (74.4 to 100.0)	68.5	73.1 (52 to 100.0)	0.78 (0.53 to 1.14)
	Breast^a^	Percentage of cohort	37.9	45.8 (21.9 to 87.8)	36.8	29.2 (11.3 to 83.2)	0.64 (0.24 to 1.71)
**Immunisation**	Number (percentage) with at least one immunisation consultation	Percentage of cohort	80.6	81.9 (70.9 to 95.5)	80.3	83.3 (67.1 to 100.0)	1.02 (0.79 to 1.30)
	Days until first immunisation consultation for those immunised	Median	39.4		95.0		
	Number of immunisation consultations for those immunised	Mean	2.5	2.5 (2.3 to 3.8)	3.5	3.3 (3.0 to 3.7)	1.30 (1.14 to 1.49)

CI = confidence intervals. RLM:QR = refugee-like migrant: quota refugee.

a Screening rates for breast screening, and cervical screening offered, calculated for eligible women aged 45–69 years and 20–69 years respectively.

For percentages, confidence interval upper limits that exceeded 100% are printed as 100% in the table (as age/sex standardisation uses a rate-based method with no upper limit).

Primary care utilisation was similar for the cohorts, except refugee-like migrants had higher primary care nurse and social work utilisation. Secondary care utilisation was very similar for the two cohorts, except for lower utilisation for refugee-like migrants for overall outpatient visits, and dental and mental health liaison contacts. Screening rates were lower for refugee-like migrants in all subcategories, but only statistically significant for tuberculosis screening. Immunisation initiation percentage was similar for the cohorts, but refugee-like migrants waited longer before initiation of immunisation, with one more immunisation consultation on average than quota refugees.

### Longitudinal healthcare utilisation

**Figure 2.**
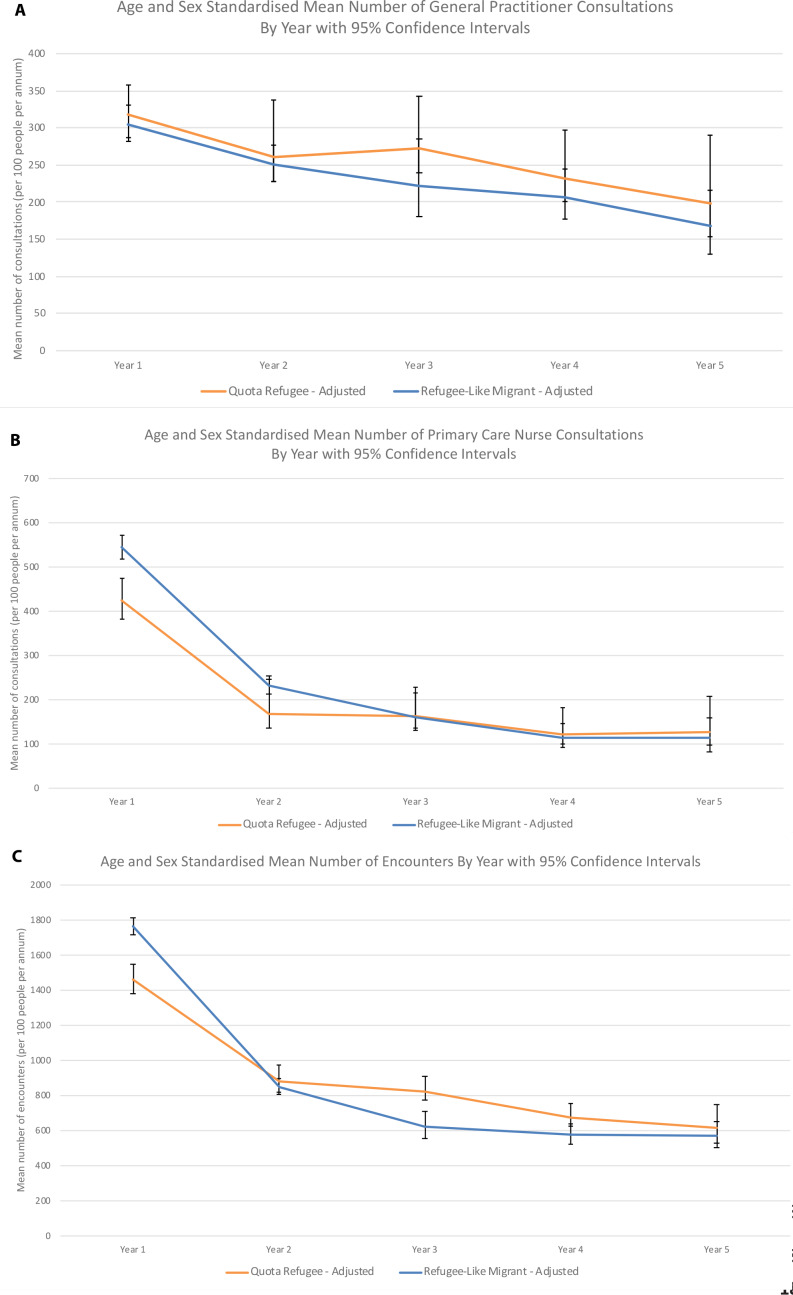
Age and sex standardised: a) GP consultations, b) primary care nurse consultations, and c) ‘encounters’, by year after arrival with 95% confidence intervals

Utilisation rates by period after arrival showed a downward trend over time. Refugee-like migrants utilised GPs at similar rates to quota refugees (Figure 2a). Refugee-like migrants utilised nurses more than quota refugees in the first 2 years, with the rates of nurse consultations for both groups dropping sharply thereafter (Figure 2b). ‘Encounters’ followed a similar trend to nurse utilisation for both cohorts.

## Discussion

### Summary

Refugee-like migrants had similar demographic profiles and similar or higher health utilisation than quota refugees in most measures, with no major changes in utilisation trends observed after age and sex standardisation. Refugee-like migrant arrivals were two-thirds the number of the quota refugee arrivals over the 5-year period. While the number of arrivals is specific to this practice, and therefore not reliably generalisable, it is reasonable to expect that wherever quota refugees are accepted, there will be a flow-on effect to refugee-like migrant family members arriving over time. This study emphasises the importance of being prepared for their health needs.

### Strengths and limitations

To the authors' knowledge, no other study has published health utilisation data for migrants with a refugee-like background despite this being an important issue for health and social care planning in countries which accept refugees. Migrants with a refugee-like background are seldom identified in health settings, so it was important to take this opportunity where an operational definition had been established and routinely used. With the complete dataset available it was possible to select all arrivals to the practice over a set period and almost all patients provided data to the end of the follow-up period. The analysis accounted for the varying length of follow-up time contributed by each participant, and enabled longitudinal analysis from time after arrival.

Healthcare access for refugee-like migrants is likely to be strongly influenced by national policy for ‘usual care’. However, the ‘usual care’ health setting and data sources for this research also introduced limitations. The research was situated within a practice-specific model of care for refugee-like migrants and quota refugees, which is not typical of other New Zealand primary care practices. Even so, the only difference in ‘usual care’ for refugee-like migrants compared with quota refugees was a focus on initiating catch-up immunisations and health screening, compared with quota refugees who had already started these as part of their systematic state-organised arrival process. Generalisability of the findings will also be affected by national policies regarding family reunification that could amplify or suppress the differences in health experiences between quota refugees and migrant family members. This might occur, for example, if there was a long delay imposed on quota refugees before allowing family reunification, or if a quota refugee sponsor was required to cover all medical costs for a subsequent migrant family member.

Additionally, refugee-like migrant groups have varied definition and interpretation in media and in research. This study used the bespoke definition created by the primary care practice for the purpose of offering clinical care to patients who had refugee-like experiences, but who were not quota refugees or asylum seekers, and this may have introduced systematic bias. It was not practical to cross-check immigration data, or to verify each participant’s actual health experiences before arrival. Thus, the health background of participants had to be inferred from description of their journey to New Zealand.

Despite covering a substantial period with high-quality data, the cohorts were relatively small and were comprised of diverse people, particularly for comparing rare outcomes or health service events, or for more detailed analysis of demographic differences such as ethnic group. This meant other health data was unable to be reliably analysed (for example, diagnoses or hospital admissions for specific conditions).

### Comparison with existing literature

There is a need to better understand how refugees and migrants affect health systems and services.^38^ Published studies have examined the health of refugee and migrant populations, and some non-health literature relating to refugees explicitly includes refugee-like migrant groups within the broader category of refugees.^39^ Analysis of humanitarian migrants’ experiences, using a concept of migration units, includes both quota refugees and family members.^40,41^ However, no literature regarding health utilisation for refugee-like migrants or potentially equivalent defined groups of migrants was found.

The study showed that refugee-like migrants and quota refugees live in deprived environments in New Zealand, which are known to be associated with poor health.^35^ Both groups showed what appeared to be high health utilisation for a population with a young age structure.^42,43^ However, the study did not directly compare with a general population cohort, and available population data was not age- or sex-standardised, and it employed different data sources and collection methods, limiting comparison. The study suggests that health and social care services should prepare for the language, complexity, and longer consultation needs of refugee-like migrants to the same extent as for refugees.^44^

Similar to other studies of refugees,^45,46^ higher initial primary care nurse utilisation (compared with GP utilisation) highlights primary care nurses’ roles in supporting initial settlement. In the study, higher nurse utilisation for refugee-like migrants compared with quota refugees may be contributed to by initial primary care nurse-led health assessment appointments and more immunisation consultations.

Social work input is very important for successful resettlement.^1^ In the study, the apparent higher primary care social work utilisation for refugee-like migrants is likely to be partially explained by quota refugees having automatic, no-cost access to Red Cross social work services after arrival;^47^ unfortunately, these Red Cross services could not be captured in the study.

Some studies show higher emergency department and inpatient and outpatient hospital use by migrants or refugees than the general population,^48,49^ and that refugees face barriers when utilising mental health services.^50^ In the current study, the similar rates of inpatient admissions suggest that refugee-like migrants and quota refugees have similar morbidity for more serious illnesses, and similar emergency department use was found. However, lower outpatient utilisation was found for refugee-like migrants compared with quota refugees*,* including for mental health consultations*,* which could be owing to morbidity differences, different health-seeking behaviour, or access barriers. Refugee-like migrants could plausibly learn health-seeking behaviour from established family members^51^ who are familiar with New Zealand health services.

A recent scoping review highlights the importance of health screening in refugee populations.^52^ In the study, screening and immunisation rates were lower for refugee-like migrants, which was likely influenced by effective health screening at the quota refugee residential orientation programme. In addition, some refugee-like migrants arrive in New Zealand under work or visitor visa categories^21^ without access to publicly funded health services. They, therefore, incur costs, and may not be eligible for national programmes. It is known that cultural sex-specific concerns about cervical and breast screening influence decisions to participate in screening,^53–55^ which might contribute to the low mammography rates that were found in both refugee-like migrants and quota refugees compared with national statistics.^56^ The tuberculosis screening did not take into account off-shore screening, which is compulsory for all long-term migrants to New Zealand, but which is repeated on arrival for quota refugees.^31^

Immunisation initiation catch-up is known to be complex.^57^ Quota refugees received their first immunisation doses at their residential orientation programme and overall rates for both groups were reassuringly similar. Eligibility status and ease of access may have contributed to delays in immunisation initiation for refugee-like migrants.

### Implications for research and practice

This study has relevance to developed countries accepting quota refugees, with special contemporary international significance because of large refugee and forced migrant movements. Former refugees exert a migration ‘pull’ to bring family members to their settlement countries outside of quota programmes. Evidence has been provided in this study that refugee-like migrants have similar or greater health utilisation to quota refugees, suggesting that refugee-like migrants need health and social care services that are systematically planned and delivered. Such planning would first require clarity of definition and ongoing health data collection for refugee-like groups, work that should be co-designed with people from refugee backgrounds.

As this is a small study, further research to determine the extent of national and international generalisability of the findings is required. Further research should investigate matched immigration and health datasets to give large scale and accurate determination of migration pathways. Qualitative research is also needed to explore factors that help or hinder the use of health systems by refugee-like migrants.
